# Predicting genome-wide redundancy using machine learning

**DOI:** 10.1186/1471-2148-10-357

**Published:** 2010-11-18

**Authors:** Huang-Wen Chen, Sunayan Bandyopadhyay, Dennis E Shasha, Kenneth D Birnbaum

**Affiliations:** 1 Department of Computer Science, Courant Institute of Mathematical Sciences, New York University, New York, NY 10003 USA; 2 Dept of Computer Science & Engineering, University of Minnesota - Twin Cities, 200 Union St SE, Minneapolis MN 55455 USA; 3 Center for Genomics and Systems Biology, Department of Biology, New York University, New York, NY 10003 USA

## Abstract

**Background:**

Gene duplication can lead to genetic redundancy, which masks the function of mutated genes in genetic analyses. Methods to increase sensitivity in identifying genetic redundancy can improve the efficiency of reverse genetics and lend insights into the evolutionary outcomes of gene duplication. Machine learning techniques are well suited to classifying gene family members into redundant and non-redundant gene pairs in model species where sufficient genetic and genomic data is available, such as *Arabidopsis thaliana*, the test case used here.

**Results:**

Machine learning techniques that combine multiple attributes led to a dramatic improvement in predicting genetic redundancy over single trait classifiers alone, such as BLAST E-values or expression correlation. In withholding analysis, one of the methods used here, Support Vector Machines, was two-fold more precise than single attribute classifiers, reaching a level where the majority of redundant calls were correctly labeled. Using this higher confidence in identifying redundancy, machine learning predicts that about half of all genes in *Arabidopsis *showed the signature of predicted redundancy with at least one but typically less than three other family members. Interestingly, a large proportion of predicted redundant gene pairs were relatively old duplications (e.g., Ks > 1), suggesting that redundancy is stable over long evolutionary periods.

**Conclusions:**

Machine learning predicts that most genes will have a functionally redundant paralog but will exhibit redundancy with relatively few genes within a family. The predictions and gene pair attributes for *Arabidopsis *provide a new resource for research in genetics and genome evolution. These techniques can now be applied to other organisms.

## Background

Plants typically contain large gene families that have arisen through single, tandem, and large-scale duplication events [1]. In the model plant *Arabidopsis thaliana*, about 80% of genes have a paralog in the genome, with many individual cases of redundancy among paralogs [2-4]. However, genetic redundancy is not the rule as many paralogous genes demonstrate highly divergent function. Furthermore, separating redundant and non-redundant gene duplicates *a priori *is not straightforward.

Mutant analysis by targeted gene disruption is a powerful technique for analyzing the function of genes implicated in specific processes (reverse genetics). Still, the construction of higher order mutants is time consuming and obtaining detectable phenotypes from knockouts of single genes generally has a low hit rate [5,6]. The ability to distinguish redundant from non-redundant genes more accurately would provide an important tool for the functional analysis of genes. Furthermore, vast public databases are now available and can be used to quantify pair-wise attributes of gene pairs to help identify redundant gene pairs [7,8].

Here we develop tools to improve the analysis of genetic redundancy by (1) creating a database of comparative information on gene pairs based on sequence and expression characteristics, and, (2) predicting genetic redundancy genome wide using machine learning trained with known cases of genetic redundancy. The term genetic redundancy is used here in a wide sense to mean genes that share some aspect of their function (i.e., at least partial functional overlap).

Different theories exist regarding the forces that shape the functional relationship of duplicated genes. One posits that gene pair survival frequently arises from independently mutable subfunctions of genes that are sequentially partitioned into two duplicate copies sometime after gene duplication, leading to different functions for the two paralogs [9-11]. However, at least some theoretical treatments show that even gene pairs that are on an evolutionary trajectory of subfunctionalization may retain redundant functions for long periods [12]. Another set of theoretical models predicts that natural selection can favor stable genetic redundancy or partial redundancy under certain conditions, especially in large populations [13,14]. Other formulations allow for simultaneous evolution of subfunctionalization, neofunctionalization, and redundancy in the same genome [15]. Thus, despite varying models on the persistence of gene duplicates, none of these formulations preclude the possibility that gene duplicates may overlap in function for long evolutionary periods.

However, a simple lack of observable phenotype upon knockout is not necessarily caused by genetic redundancy. Other causes include 1) phenotypic buffering due to non-paralogous genes or network architecture [16] 2) minor phenotypic effects in laboratory time scales but major effects over evolutionary periods [17,18], or, 3) untested environments or conditions in which a gene is necessary [19]. This report is focused exclusively on redundancy through functional overlap with a paralogous gene.

Thus, for the sake of training our methods, redundant gene pairs are defined as paralogous genes whose single mutants show little or no phenotypic defects but whose double and higher order mutant combination, when available, show a significant phenotype. Thus, such gene pairs are redundant with respect to an obvious phenotype. Genes that show single mutant phenotypes were used as a negative training set. These genes, together with their closest BLAST match in the genome, comprised the non-redundant gene pairs, a conservative bias against over-fitting on BLAST statistics. The training set consisted of 97 redundant and 271 non-redundant pairs for *Arabidopsis*, which were compiled from the literature. Preliminary data showed that the redundant and non-redundant sets possessed distinct properties with respect to pair-wise attributes of gene duplicates.

Training sets can be used to learn rules to classify genetic redundancy, using common properties, or attributes, of gene pairs. The attributes compiled for this study compare different aspects of nucleotide sequence, overall protein and domain composition, and gene expression. Since any gene pair can be compared using the same common attributes, these rules can then be applied to unknown cases to predict their functional overlap.

A set of "rules" for redundancy can be generated by machine learning, which uses the attributes of known examples of positive and negative cases in training sets to classify unknown cases [20]. Machine learning has been applied to a range of biological problems [21], including the prediction of various properties of genes such as function or phenotype [22-25] and network interactions [26]. In *Arabidopsis*, sequence expression attributes of individual genes have been used to predict gene function [27]. Here a new dataset was compiled to test the novel question of learning the signatures of genetic redundancy on a genome-wide scale.

Here we show that predictions based on a Support Vector Machine achieved a precision of about 62% at recall levels near 50%, performing two-fold better than single attribute classifiers, according to withholding analysis. This performance is better than expected because positive examples are plausibly rare among all family-wise gene pairs and the causes of redundancy are apparently complex. The level of precision achieved permits reasonable estimates of trends in redundancy at a whole-genome level. The predictions show that more than 50% of genes are redundant with at least one paralog but typically no more than three in the genome. In many cases, the method predicts that redundant gene pairs are not the most closely related in a gene family. Together, the results show that redundancy is a relatively rare outcome of gene duplication but any given gene is likely to have a redundant family member. This appears partly due the property that redundancy persists or re-establishes itself for complex reasons, meaning not only due to the age of a gene duplicate. For example, many redundant duplicate pairs appear to be greater than 50 million old, according to estimates based on synonymous substitution rates. In addition, gene pairs from segmental duplications have a dramatically higher probability of redundancy and certain functional groups, like transcription factors, show a tendency to diverge. The entire dataset, including attributes of gene pairs and SVM predictions is available at http://redundome.bio.nyu.edu/supp.html.

## Results and Discussion

### Training set evaluation

A threshold question in this study is whether a gene pair can be reliably labeled as redundant or non-redundant in the training set, given that different gene pairs often have different phenotypes. Preliminary analysis showed that select attribute values had distinct distributions between the two groups. For example, BLAST E-values were, in general, lower in the redundant pairs than in the non-redundant pairs in the training set, indicating they share higher sequence similarity (Additional file 1, figure a). A similar trend held for non-synonymous substitution rates between the two groups (data not shown). Similarly, on average, gene pairs in the positive training set exhibited higher expression correlation levels over the entire dataset (R = 0.51) than gene pairs in the negative training set (R = 0.28). Thus, known redundant gene pairs appear to have a higher correlation than gene pairs identified as non-redundant, as expected (Additional file 1, figure b). The disparate trends in the two groups of gene pairs sets do not prove that all training set examples are correctly labeled or that all gene pairs can be discretely labeled but it does indicate that the genes labeled redundant, in general, show distinct attributes from those labeled non-redundant. Thus, there is a basis for asking whether combinations of gene pair attributes could be used to improve the prediction of genetic redundancy.

### Algorithm Choice

**Figure 1:**
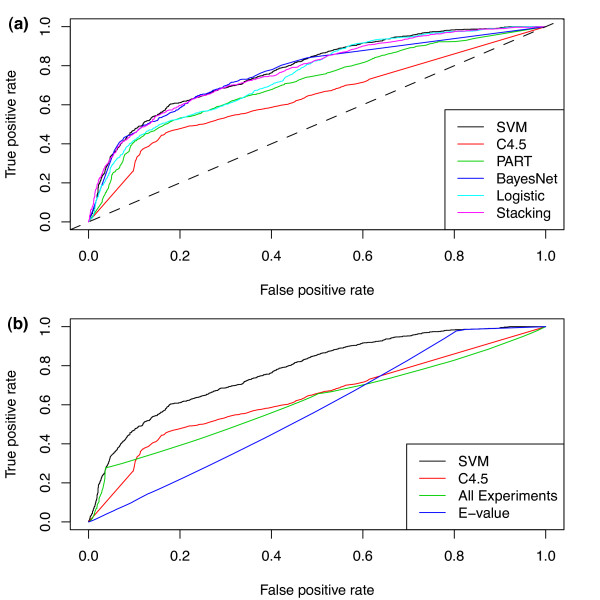
**Performance analysis of machine learning and single attribute classifiers**. Receiver Operating Characteristic (ROC) curve for comparing (A) 5 different machine learning algorithms and one meta-algorithm (StackingC); The hashed diagonal line is the performance of a simple betting classifier, which represents probabilistic classification based on the frequency of positive and negative cases in the training set. (B) single-attribute classifiers using correlation of gene pairs across all microarray experiments (All Experiments) and BLAST E-values.

Instead of predicting binary labels for genes pairs, machine learning methods can quantify redundancy by posterior probabilities, which permit performance evaluation at different levels of confidence. The Receiver Operating Characteristic (ROC) curve (Figure 1a), which plots true *vs*. false positive rates at all possible threshold values, shows that SVM, Bayesian network, and stacking (a combined method) performed better than decision trees, decision rules, or logistic regression. All machine learning algorithms dramatically outperformed a random, or betting, classifier (Figure 1a, diagonal line), which also supports the hypothesis that the training set labels are not randomly assigned. SVM was used for further analysis because of its good empirical performance and well-characterized properties [20].

The ROC curve analysis also permits an evaluation of an appropriate threshold for calling redundant vs. non-redundant gene pairs. Using precision (true positive rate among positive calls) and recall (true positives among positive calls vs. total true positives), the precision rate increased relatively sharply from 0.2 to 0.4 probability. The rate then saturated after 0.4 while recall dropped sharply after that point (Additional file 2). Thus, 0.4 was chosen as a balanced tradeoff between true and false positives for further analysis of SVM.

### Machine learning performance

Another critical question is whether machine learning, which considers multiple features of duplicate gene pairs, offers any advantage over the single characteristics commonly used by biologists to identify potentially redundant genes, such as sequence similarity or expression correlation. To address this question, predictions for single attributes were generated using information gain ratio. A 10-fold withholding approach was used to evaluate performance. ROC curves (Figure 1b) showed that sequence similarity and expression correlation, taken individually, have poorer performance than SVM or Decision Trees. The area under the curve (AUC) and above the non-discriminating line measures performance over random guessing (0 to 1 scale). The AUC was 0.56 for SVM while the AUC for BLAST E-values and correlation was 0.14 and 0.22, respectively. At every threshold cutoff, SVM outperformed single characteristic approaches.

Within gene families, the vast majority of pair-wise combinations of genes within gene families are expected to be non-redundant. In such a problem, a classifier could perform well (but not usefully) by labeling all comparisons as functionally non-redundant. Thus, a critical feature of a useful predictor is achieving a good performance on redundant cases.

To evaluate directly the tradeoff between accuracy and coverage, we compared precision and recall among the different classifiers. At the 0.4 probability cutoff established for SVM, the machine learning approach achieved a precision of 0.62 with a recall of 0.48. In contrast, at the same recall rate, expression correlation achieved a precision of 0.36 and BLAST E-values a precision of 0.29. Thus, in addition to ROC curve analysis, the machine learning approach that utilized multiple attributes showed dramatically improved precision in labeling redundancy compared to using single attributes.

SVM also performed well on predicting non-redundant gene pairs at the 0.4 probability threshold, with a precision rate of 0.83 and a recall rate of 0.90. At the same recall level, expression correlation has a precision of 0.82 and BLAST E-values had a precision of only 0.25. Thus, the SVM classifier shows consistently high recall on negative cases with moderate levels of precision and recall on the difficult task of identifying scarce positive examples.

When tested with 16 new redundant and 9 non-redundant pairs published after the initial training of the predictor, the SVM classifier predicted 11 pairs were redundant based on the 0.4 probability threshold, with 10 of them true redundant cases. The precision on positive cases was >90% with a recall of about 63%. The precision on negative cases was 57% with a recall of 89%. Thus, the classifier performs well on novel cases not used in withholding analysis.

### The scale of predicted redundancy

At a probability score greater than or equal to 0.4, SVM predicted 16,619 redundant pairs among 593,673 (2.80%) pair-wise comparisons taken from genes that fell into annotated or *ad-hoc *gene families (see Materials and Methods). The percentage of redundant pairs at various probability scores is shown in Additional File 3. At the 0.4 cutoff, 8,628 out of 18,495 genes examined, or an estimated 47% of the genes tested, were predicted to exhibit high levels of redundancy with at least one other gene. Extrapolating estimates of true and false positive rates at this probability, about 11,000 genes, more than half the large set of genes tested, are predicted to have a highly redundant paralog. Nonetheless, the number of redundant genes is likely an underestimate since 4,757 genes could not be evaluated for redundancy because they were not on the ATH1 microarray. Many of the missing genes are likely to be closely related so this set may show higher redundancy rate than the background.

**Figure 2:**
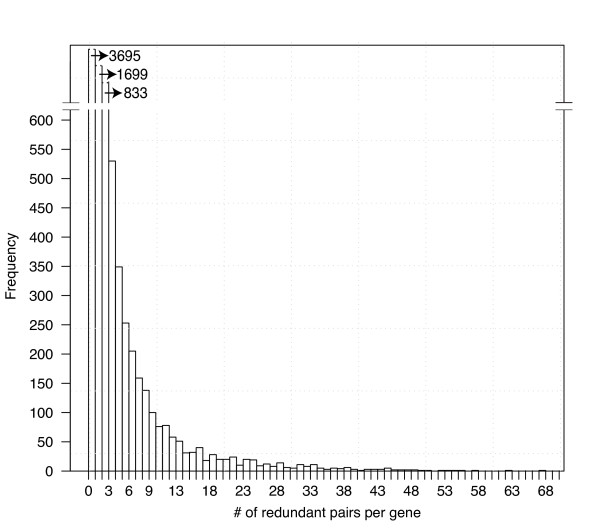
**The predicted depth of redundancy genome-wide**. Genes are grouped into bins based on the number of paralogs with which they are predicted to be redundant. The first bin represents the number of genes that were predicted to have exactly one redundant paralog, using the cutoff of 0.4. The frequency distribution shows that most genes have relatively few predicted redundant duplicates.

Among the 8,628 genes classified as redundant, many were labeled redundant with more than one paralog. However, the frequency distribution of redundant paralogs per gene is skewed to the left, meaning that the largest categories are genes with relatively few redundant paralogs (Figure 2). For example, the largest category (3,695 or about 43% of redundant genes) were predicted to have only one redundant duplicate. The majority of redundant genes (5,394 or 63%) were predicted to have no more than two duplicates. While the false negative rate may mean that many duplicate pairs were not detected, the general trend indicates that most genes tend to have relatively few redundant genes associated with them. Overall, these predictions suggest that redundancy in gene function is common in the *Arabidopsis *genome but the number of functionally redundant genes for any given trait is relatively low.

The synonymous substitution rate (K_s_) of gene pairs was used to roughly examine the age of gene duplications. As expected, redundant gene pairs had lower synonymous substitution rates on average, meaning that redundant gene pairs tended to be younger duplicates (Additional File 4). However, the frequency distribution of predicted redundant gene pairs plotted against K_s _has a slow decline and a long tail, suggesting that many redundant pairs are quite old. For example, 41% of redundant pairs have a K_s _> 1, which is commonly estimated to exceed 50 million years [4]. Thus, predictions indicate that redundancy can persist for long evolutionary periods.

### How attributes contribute to predictions

Given that multiple attributes improve the predictability of redundancy, we asked which attributes contribute most to predictions. Two measures were used to assess the informativeness of individual attributes on SVM predictions: 1) the absolute value of SVM weights, which are the coefficients of the linear combination of attributes that is transformed into redundancy predictions (see Materials and Methods), and, 2) SVM sensitivity analysis in which single attributes were removed and the overall change in predictions was quantified (using correlation of probability values compared to the original SVM predictions). The two analyses were largely in agreement in identifying the top ranking attributes (Additional file 5) and an average of the two ranks was used as a summary rank.

Several unexpected attributes ranked highly in the analysis, suggesting that functional information on gene pair divergence could be captured by attributes that are rarely utilized. The highest-ranking attribute was "isoelectric point" (Rank 1), which measures a difference in the pH at which the two paralogs carry no net electrical charge. Thus, the measure is sensitive to differences in the balance of acidic and basic functional groups on amino acids, potentially capturing subtle functional differences in protein composition. Similarly, "Molecular Weight" ranked fifth. An index of the difference in "Predicted Protein Domains" ranked seventh, apparently providing functional information on the domain level.

It was noteworthy that typically summary statistics on protein or sequence similarity did not rank highly. For example, BLAST "Score" (Rank 17), "E-value" (Rank 23), and "Non-Synonymous Substitution Rate" (Rank 28) were not among the top ranking attributes, although preliminary analysis showed they contained some information pertaining to redundancy. The low contribution of these attributes was partly due to the fact that gene pairs were already filtered by moderate protein sequence similarity (BLAST E-value of 1e-4, see Materials and Methods) but this cutoff is relatively non-stringent. Thus, measures that capture changes in protein composition like isoelectric points or predicted domains appear more informative about redundancy at the family level than primary sequence comparisons.

For gene expression, two types of experimental categories had a high rank for predictions, those that contained many experiments and those that examined expression at high spatial resolution. In the first category, "All Experiments" (Rank 4), "Pathogen Infection Experiments" (Rank 6), and "Genetic Modification Experiments" (Rank 7) were among the top ranked categories. These categories all shared the common feature of being among the largest, comprised of hundreds of experiments each (Table 1). Thus, large datasets appear to sample enough expression contexts to reliably report the general co-expression of two paralogs for redundancy classification. The specific experimental context may also carry information.

**Table 1:** List of attributes used for the predictions

#	Attribute	Type	Description
1	CLUSTALW Score	Sequence	ClustalW alignment score

2	E-value	Sequence	BLAST alignment E-value

3	Isoe Pt Diff	Sequence	percent difference in isoelectric points

4	Mol W Diff	Sequence	percent difference in molecular weight

5	Nonsyn Subst Rate	Sequence	non-synonymous substitution rate

6	Protien Domain Sharing Index	Sequence	intersection/union of predicted protein domain

7	Score	Sequence	BLAST alignment bit score

8	All Experiments	Expression	2799 ATH1 microarray experiments

9	Atlas of Arabidopsis Development	Expression	264 ATH1 microarray experiments

10	Atmosphereric Conditions	Expression	172 ATH1 microarray experiments

11	Change Light	Expression	127 ATH1 microarray experiments

12	Change Temperature	Expression	112 ATH1 microarray experiments

13	Compound Based Treatment	Expression	248 ATH1 microarray experiments

14	Genetic Modification	Expression	952 ATH1 microarray experiments

15	Genetic Variation	Expression	22 ATH1 microarray experiments

16	Growth Condition Treatments	Expression	74 ATH1 microarray experiments

17	Growth Conditions	Expression	503 ATH1 microarray experiments

18	Hormone Treatments	Expression	256 ATH1 microarray experiments

19	Induced Mutation	Expression	18 ATH1 microarray experiments

20	Infect	Expression	61 ATH1 microarray experiments

21	Injury Design	Expression	28 ATH1 microarray experiments

22	Irradiate	Expression	28 ATH1 microarray experiments

23	Light	Expression	12 ATH1 microarray experiments

24	Media	Expression	54 ATH1 microarray experiments

25	Organism Part	Expression	806 ATH1 microarray experiments

26	Organism Status	Expression	16 ATH1 microarray experiments

27	Pathogen Infection	Expression	200 ATH1 microarray experiments

28	Root Cells	Expression	59 ATH1 microarray experiments

29	Root Cells Iron Salt Treatments	Expression	17 ATH1 microarray experiments

30	Root Cells Nitrate Treatments	Expression	20 ATH1 microarray experiments

31	Root Developmental Zones	Expression	11 ATH1 microarray experiments

32	Root Developmental Zones (Fine Scale)	Expression	24 ATH1 microarray experiments

33	Root Regeneration	Expression	11 ATH1 microarray experiments

34	Seed Development	Expression	6 ATH1 microarray experiments

35	Set Temperature	Expression	4 ATH1 microarray experiments

36	Starvation	Expression	22 ATH1 microarray experiments

37	Stimulus or Stress	Expression	320 ATH1 microarray experiments

38	Strain or Line	Expression	32 ATH1 microarray experiments

39	Temperature	Expression	15 ATH1 microarray experiments

40	Time Series Design	Expression	427 ATH1 microarray experiments

41	Unknown Experimental Design	Expression	8 ATH1 microarray experiments

42	Wait	Expression	17 ATH1 microarray experiments

43	Water Availability	Expression	40 ATH1 microarray experiments

In contrast to providing information over a broad range of experiments, tissue and cell-type specific profiles had relatively few experiments but appeared to provide useful information on a fine spatial scale. For example, "Root Cells," which is a compendium of expression profiles from cell types [28,29], ranked 2^nd ^for all attributes. At organ level resolution, "Organism Part" ranked 11^th^[30]. Similarly, the large-scale and spatially resolved expression data sets were not highly correlated to each other (e.g., "Pathogen Infection" and "Root Cells," R = 0.19). Thus, while attributes are not completely independent, they appear to provide different levels of information that machine learning can use to create a complex signature to identify redundancy.

In one example, four members of the *PLETHORA *(*PLT*) family have been shown to be redundant in root development [31]. One member of the family, *PLT2 *(AT1G51190), was in the training set with redundant relationships specified between it and *PLT1 *(AT3G20840) and *PLT3 *(AT5G10510). However, our classifier also correctly predicted redundancy with a fourth member of the family, *BABYBOOM *(*BBM*, AT5G17430), which was not in the training set but has been shown to be at least partially redundant with *PLT2 *for embryonic phenotypes [31]. Interestingly, even though several members of *PLT *family are more closely related in sequence to *PLT2 *than *BBM*, they were not predicted to be redundant (to date no redundancy among them and *PLT2 *has been documented). Thus, this example shows the properties of useful predictor for genetic redundancy, accurately delineating redundancy within a gene family in a non-trivial way; that is, the predictor does not simply label the most closely related genes as redundant.

In addition, we note that high probability predictions for redundancy (or non-redundancy) can be based on different sets of attributes. For example, while redundant pairs tended to have high gene expression correlation, many gene pairs classified as redundant have low correlation levels (~20% of them have R < 0.2 in "All Experiments," see Additional file 1, figure b). In such cases, other attribute values that generally suggested redundancy influenced the redundancy prediction. Additionally, we also generated separate predictions based on co-expression in specific categories of experimental conditions, such as root cell types or nutrient environments, leaving out overall trends in correlation across all experiments. Such techniques could specifically address partial redundancy in conditions where a sufficient number of global gene expression experiments were available (online supplementary data, http://redundome.bio.nyu.edu/supp.html). Thus, one advantage of machine learning approaches using multiple attributes is that they can discover multiple combinations of attributes that suggest redundancy or non-redundancy.

### Functional trends in predicted genome-wide genetic redundancy

The ability to identify redundancy at reasonable accuracy across the genome permits an analysis of genome-wide trends in the divergence of gene pairs. Gene Ontology was used to ask whether certain functional categories of genes were more likely to diverge or remain redundant according to predictions. To control for the number of closely related genes, paralogous groups of genes were binned into small (< 5), medium (≥ 5,≤ 20,) or large (> 20) classes based on the number of hits with a BLAST cutoff of 1e-4 or less. In each class, gene pairs were split into redundant and non-redundant categories and each group was analyzed for over-represented functional categories (see Materials and Methods).

**Figure 3:**
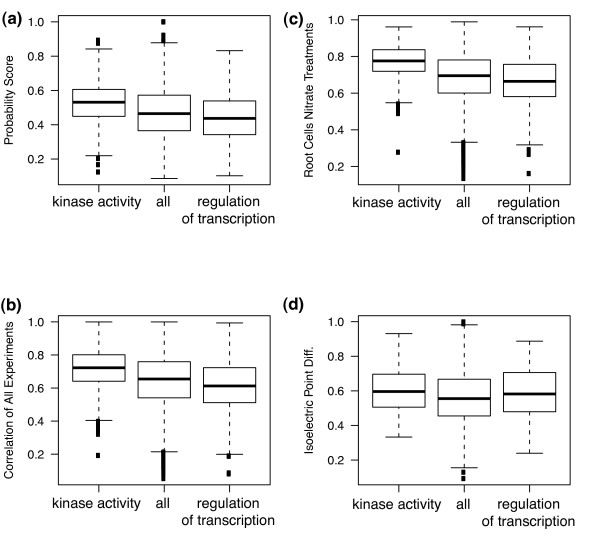
**Trends in redundancy predictions and attributes in different functional categories**. Box and whisker plots show landmarks in the distribution of values, where the horizontal line represents the median value, the bottom and top of the box represent the 25^th ^and 75^th ^percentile values, respectively, and the whisker line represents the most extreme value that is within 1.5 interquartile range from the box. Points outside the whisker represent more extreme outliers. The category "all" represents all genes in the large size class (see text) and is used as a background distribution. The two other categories represent genes in the GO functional category named.

We focused analysis on signal transduction since such genes are the frequent targets of reverse genetics and distinct trends in these categories emerged from the data. Within this large-sized paralog group, non-redundant genes were over-represented in the category of regulation of transcription (p < 10^-6^, 301 genes, Additional file 6). These included members of the AP2-EREBP (52), basic Helix-Loop-Helix (33), MYB (35), MADS-box (18), bZIP (16), and C2H2 (10) transcription factor families (Additional file 7). Similarly, in the small-sized families, the same term was also over-represented among non-redundant genes (p < 0.01) with subgroups of many of the same gene families mentioned above. In the large gene family class, the frequency of transcriptional regulators with at least one redundant paralog was only 30% compared to a background of all genes with 50%. Similar trends were observed in the distribution of predicted probabilities of redundancy, with transcription factors skewed toward lower values (Figure 3a)

We examined the average values of attributes among redundant and non-redundant pairs to ask how attribute values contributed to classifications. On the gene expression level, gene pairs in the transcriptional regulator category showed a distribution of correlation values that was skewed toward lower values (e.g., Figure 3b, c). For sequence attributes, transcriptional regulator gene pairs also showed higher differences in isoelectric points compared compared to all genes in large family size class (Figure 3d). In summary, transcriptional regulators in large gene family classes show a trend of functional divergence of gene pairs, with tendencies to diverge in expression pattern and in subtle protein properties.

In contrast, genes predicted to be redundant were dramatically over-represented in the category of kinase activity, another category of signal transduction (p < 10^-51^, 817, Additional file 6). The term included many members of the large receptor kinase-like protein family (Additional file 7). The distribution of redundancy probability for gene pairs in the kinase category was skewed toward higher values compared to all genes in the same large family class (Figure 3a). In contrast to the transcriptional regulator category, about 85% percent of kinases had at least one predicted redundant paralog. In general, the redundant kinases show the typical trends of redundant genes from other categories, with high correlation over a broad set of experiments (Figure 3b, c). Interestingly, despite the high level of predicted redundancy, gene pairs in the kinase category also showed a high divergence in isoeletric points (Figure 3d), showing that this attribute trend of kinases, which typically signified non-redundancy, was overcome by other attributes. Overall, the redundancy analysis suggests that genes at different levels of signal transduction show distinct trends in redundancy, which has intriguing implications for the general role of different signaling mechanisms in evolutionary change.

It is important to note that not all attributes showed the same trends in each functional category. For example, other functional categories with redundant genes showed different trends in attributes relative to the background, as noted above for the isoeletric point attribute for kinases. This means that, as noted above, the attributes display some independence and machine learning can rely on different attributes to call redundancy in different genes.

### Duplication Origin and Predicted Redundancy

We also asked whether there were trends in redundancy stemming from either single or large-scale duplication events. To compare redundancy trends by duplication origin, gene pairs were labeled according to previous genome-wide analyses that identified recent segmental duplication events in *Arabidopsis *[32] as well as tandem and single duplications [33]. To minimize bias that might be caused by a correlation with the age of a duplication event, only gene pairs with a synonymous substitution rate (K_s_) below 2 were used. The cutoff, in addition to the fact that many very recent duplicates were not included on the microarray and could not be analyzed, made the distribution of K_s _values in recent and single duplication events highly similar (Additional file 8a, b). Thus, the comparison of these two groups was not confounded by differences in the apparent ages of duplication events in the recent segmental vs. single duplication events.

The average probability of redundancy was significantly higher among gene pairs in the most recent duplication event than among gene pairs resulting from single duplication events (0.47 v.s. 0.28, p < 10^-15 ^by t-test). Despite the equilibration of neutral substitution rates, gene pairs in the two groups differed dramatically, on average, in molecular weight difference (0.04 recent vs. 0.13 single) and isoelectric point difference (0.8 recent vs. 0.12 single). In addition, expression correlation between gene pairs was generally two-fold higher in recent duplicates than in single duplication events. Higher predicted redundancy among segmental duplicate pairs was not trivially due to larger gene families in that class, as the number of closely related genes for gene pairs in the single, old, recent, and tandem events is 54, 7, 20, and 46, respectively. It is possible that synonymous substitution rates do not accurately reflect relative divergence times but it is not apparent how one group would show bias over the other. Thus, the predictions suggest that duplicates from large segmental duplications diverge more slowly in function, as is evident in low divergence in expression and protein-level properties.

## Conclusions

Identifying redundancy is a complex problem in which gene pairs may be redundant in some phenotypes but not others. However, the results indicate that there is enough generality in the outcome of gene duplication to classify redundancy based on evidence from disparate phenotypes. Among the gene pairs that the SVM classified as redundant, 62% were correct in withholding analysis. At this level of precision for redundancy predictions, SVM was able to correctly label 48% of all known cases of redundant gene pairs. The best single attribute classifier achieved a precision of only 36% at a cutoff that correctly labeled 48% of known cases of redundancy. The ROC curve analysis showed that no single attribute classifier performed better than SVM at any point in the analysis of true positives vs. false positives. Overall, the performance of the machine learning using multiple attributes was about twice as high as single attribute methods. The ability to predict redundancy at reasonable precision and recall rates constitutes a resource for studying genome evolution and redundancy in genetics.

### Informative attributes

The strength of the machine learning approach is that it can take advantage of multiple types of information and give each type a different weight. While more than 40 attributes were used in the analysis, the effective number of gene pair attributes was likely much smaller due to correlations among attributes. However, four or five distinct sets of attributes showed low correlation to each other and were shown to be informative for classification.

Among attributes related to sequence composition, the most informative ones were not those typically used to assess genetic redundancy. The highest-ranking attribute was an index of the difference in isoelectric points. The fifth highest-ranking attribute was an index of the difference in molecular weights. An index of predicted domain sharing also ranked highly, largely because results were sensitive to its removal from the machine learning process, indicating that it provided relatively unique information. It was surprising that BLAST E-values provided little information at values lower than 1e-4, the cutoff for the pairwise comparisons. This implies that, within gene families, other measures such as changes in the charge, composition of proteins or alterations in the domain structure are better indicators of functional redundancy than direct sequence comparison metrics.

Two types of expression-based attributes were informative, including those comprised of many experiments and those that resolved mRNA localization into specific tissues or transcriptional response to an environmental stimulus. While the large expression datasets were highly correlated (e.g., "Genetic Modification" and "Organism Part, R = 0.81), they were much less correlated with the high-resolution data (e.g., "Root Cells" and "Genetic Modification", R = 0.30). Thus, it appears that different types of expression data are contributing at least some distinct information, with high spatial resolution datasets providing informative contextual information and larger datasets tracking the broad behavior of duplicate genes.

### Functional trends in redundancy

Genes annotated with roles in transcriptional regulation, including many transcription factors, showed a tendency toward functional divergence. The opposite trend occurred at another level of signal transduction with kinases showing a tendency toward redundancy. Interestingly, divergent redundant transcriptional regulators showed, on average, a divergence in isoelectric points compared to the background or even other redundant categories. This global trend fits arguments, based on case studies, that modular changes in transcription factor proteins are plausible mechanisms for evolutionary change [34]. For example, it has been postulated that subtle changes in proteins such as insertions of short linear motifs that mediate protein-protein interactions and simple sequence repeats of amino acids could play a role in functional divergence of transcription factors outside of dramatic changes to the DNA binding site[35,36]. Still, a more systematic examination of protein interactions among transcription factors is needed to corroborate these findings. In general, the ability to classify large groups of genes enables an analysis of the functional trends that shape redundancy in a genome.

### Implications for Genome Organization

The high level of redundancy predicted in this study is in accordance with low hit rates in reverse genetic screens in *Arabidopsis *and the high number of studies that have shown novel phenotypes in higher order mutants. However, the estimated redundancy rates still leave room for other explanations to account for the lack of single mutant phenotypes. For example, the machine learning approach predicted that 50% of genes are not buffered by paralogous redundancy but reverse genetic screens rarely achieve such a high rate of phenotype discovery. The predicted redundancy rate may be an underestimate, as about 23% of all gene pairs identified in the study could not be analyzed. Still, one implication of our results is that other prevalent phenomenon are likely to buffer gene function including, for example, network architecture or non-paralogous genes. Machine learning could eventually be applied to these other forms of redundancy but a comprehensive training set for these phenomena is currently lacking.

While the machine learning approach predicted that half the genes in the genome had a redundant paralog, most genes had no more than two other highly redundant paralogs. This leads to the paradoxical conclusion that, while the function of many or even most genes is buffered by a redundant paralog, redundancy is a relatively rare outcome of gene duplication. In addition, the forces that shape redundancy appear to be complex and not strictly a function of time. For example, a large proportion of predicted redundant gene pairs were quite ancient in their origin. And, the mode of duplication, by either single or large segmental duplication, also strongly influenced the tendency for gene pairs to diverge, according to predictions. Together, these findings suggest that redundancy between pairs is a relatively rare but targeted phenomenon with complex causes, including mode of duplication, time, and gene function.

### Implications for Genetic Research

From a practical standpoint, SVM predictions still carry enough uncertainty of false positive and false negative calls that they should be considered a guide to be used with researcher knowledge rather than a certain prediction. We envision that geneticists who are already interested in conducting reverse genetic studies of a gene of interest will often want to explore the possibility of redundancy within the same gene family. The gene of interest can then be queried in our predictions to first evaluate the number of predicted redundant genes. A large number of predicted redundant genes may be grounds for prioritizing another gene. If a small number of gene family members are implicated in redundancy and single mutants fail to display a genotype, researchers can use predictions to guide the construction of double or higher mutants. Quite often the most sequence-similar gene is not the one predicted to most likely be redundant.

In the future, predictions can be improved by having more training data to learn redundancy in more narrowly defined phenotypes. In addition, a more objective and quantitative definition of redundancy would likely improve the quality of the training set. For example, the set of downstream targets for transcription factors could provide a standardized quantitative measure for single and double mutant phenotypes. These types of data would require significant work from any individual research group. However, the training set is continuously under expansion due to the efforts of the genetic research community as a whole. Studies investigating direct targets of transcription factors are also increasingly common. Thus, the predictions of the machine learning approach will improve over time. We view this report as a first generation approach to exploring the genome-wide outcomes of gene duplications using machine learning approaches, where reasonable predictions are now feasible.

## Methods

### Defining Gene Families

We used gene family annotations available through The Arabidopsis Information Resource (TAIR) [37] which included 6,507 genes in 989 families. To group genes that were not annotated into gene families in TAIR, we established "ad-hoc" gene families, in which all members had at least one member in the family with a protein-protein BLAST E-value lower than 1e-4 and no members appear in the annotated families. Among genes for which we generated predictions, there were a total of 17,158 genes grouped into ad-hoc gene families. We did not make predictions on the singletons or genes lacking a probe on the ATH1 microarray. Thus, these genes were removed from the analysis. After this step, there were 5,644 genes in the annotated families and 12,851 genes in the ad hoc gene families.

### Attribute Data Sources and Comparative Measures

For **expression based characteristics**, we downloaded all available microarray experiments from Nottingham Arabidopsis Stock Centre (NASC)[8] for the ATH1 microarray. We further partitioned these experiments using the categorical ontology developed by NASC using the MGED classification as found in the Treeview section in NASC. If two or more partitions overlapped by more than 50 percent, we eliminated the smaller partition. We created additional partitions using data from several different cell type-specific profiling experiments [38-40], root developmental zones [28], fine-scale root developmental zones [29], dynamic profiling of root cells under treatment with nitrogen [41], and root cells responding to abiotic stress [42]. Pearson correlation was used to compare gene expression of gene pairs in each partition separately. Gene expression was normalized by MAS5 [43], a normalization method that appears to have less likelihood of spurious inter-array Pearson correlation as described in [44].

For sequence based attributes, we used TAIR protein sequence to generate pairwise attributes for gene duplicates on protein BLAST E-values, BLAST scores, and ClustalW alignments[45]. We also calculated non-synonymous substitution rates using PAML [46]. The predicted domain sharing index was based on the intersection/union of predicted domains for each protein pair, where the predicted domains were downloaded from TAIR. We also used percent difference in isoelectric points where values for each protein were downloaded from TAIR. To remove redundant attributes, we manually selected the subset in which all the pair-wise Pearson correlations between attributes in the subset are lower than 0.85.

For the on-line supplementary data, predictions were derived from either using all attributes or subsets of the data for assessing redundancy in specific biological contexts. When subsets of the data were used, all sequence attributes were used but in combination with only sets of microarray data that corresponded to biological categories, such as stress, hormone treatment, root cell type expression profiles, or light manipulation. The training set that consists of all the attributes is also available in Additional file 9, and redundancy predictions are available in Additional file 10.

### Description of Machine Learning Programs

We tested six different machine-learning programs and selected Support Vector Machine (SVM) for detailed analysis, based on the principle of Occam's razor [47]. All programs were compared using Weka's implementation [48]. For SVM, we used Weka's wrapper for LibSVM [49] for performance evaluation but used LibSVM directly when predicting functional overlap. Below is a brief summary of each:

**Decision trees **involve creation of a tree (often bifurcating) in which each tree node specifies an attribute and a threshold to choose a decision path. A particular instance of the data (e.g. gene pair) is mapped starting from the root and proceeding until a leaf is reached. Each leaf contains a specific label (e.g. overlapping or non-overlapping function). At each node in the decision tree, the gene pair is interrogated about its value on a specific attribute (such as expression correlation in a particular experiment). Thus, the path through the tree depends on the specific attributes of the gene pair. We used Weka's C4.5 [50] implementation to generate the decision tree from the training set. For each attribute, the algorithm selects the threshold that maximally separates the positive and negative instances in the training set by using the information gain measure. Therefore, decisions are taken sequentially until a terminal leaf is reached. The label of the leaf is determined by the majority rule of labels from the training set. We set the PruningConfidenceFactor to 0.25 (to address overfitting in the training set) and minNumObj to 2.

**Decision rules **specify conditions that must simultaneously be satisfied in order to assign a label. Given a list of decision rules, these rules are tested sequentially until a label is assigned, or otherwise the default label applies [51]. PART [52] was used to learn the decision rules from the training set. It learns a rule by building a decision tree on the current subset of instances, converting the path from root to the leaf that covers the most instances into a rule. It then discards the tree, removes the covered instances, and learns the next rule on the remaining instances. We used Weka's implementation of PART with the parameters PruningConfidenceFactor set to 0.25 and minNumObj set to 2.

**Bayesian network **is a generalized graphical model that assigns probabilities to specific labels. Bayesian networks model conditional dependencies as the network topology: in this network, attributes and the label are modeled as nodes and their conditional dependencies are specified by directed edges. Each node also stores a probability table conditioned on its parent nodes. The probability for a label is proportional, based on Bayes rule, to the joint probability density function of all attributes and the label, which is further decomposed into the product of conditional probability of each node given its parents. We used K2 [53] to learn the network structure. It employs a hill-climbing strategy to iteratively refine the network structure by adding directed edges and maximizing the likelihood such that it best describes the training data. We used Weka's implementation of K2 with the parameter MaxNrOfParent set to 1, which essentially restricts the learned network to be Naive Bayes [54].

**Logistic regression **uses a statistical model that assumes a linear relationship among attributes[55]. It uses the logistic function that relates the linear combination of attributes to the probability of the label. One way to learn the coefficients in the linear equation is to maximize the log-likelihood function that estimates the fitness between the predicted probability and the actual label specified in the training data. We used Weka's implementation and default parameters.

**Stacking **(StackingC) [56] is a meta algorithm, which makes prediction by combining the predictions from the participating machine learning algorithms. StackingC employs a linear regression scheme to merge the predictions: the final predicted probability of a label is the linear combination of the probabilities predicted by participating algorithms; in other words, it is a weighted average of predictions where the weights for participating algorithms were learned from the training set through a nested cross-validation process. We used Weka's implementation of StackingC to combine predictions from decision trees, decision rules, Bayesian network, logistic regression, and SVM.

**Support vector machine (SVM) **predicts the label of each instance by mapping it into a data point in a high dimensional space, whose coordinates are determined by the values of attributes [57]. The hyperplane is learned from the training set such that it separates instances with different labels and also maintains the maximum margin to the nearest data points. A test case is then labeled functionally overlapping or non-overlapping depending on which side of the hyperplane it falls. One important property of maximum margin is that the error rate, when generalized to all the data points from the sample space, is mathematically bounded. Furthermore, through the use of a kernel function, points can be transformed non-linearly into a higher or even infinite dimensional space where a better separating hyperplane might exist. We used LibSVM [49] with linear kernel and default parameters. We tested a range of the penalty parameter C (from 10^-5 ^to 10^3 ^with the log-scaled interval) and found performance to be robust to a range of settings for C. We used the default parameter (C = 1), which had the best performance. Attributes were normalized to 0[1] before learning and prediction.

Platt's probabilistic outputs for SVM provide a quantitative way for the confidence of redundancy predictions [58]. This calibrated posterior probability for the redundancy label is based on the distance from each data point to the hyperplane: larger distances on the redundant side of the hyperplane result in larger probabilities, and similarly, larger distances on the non-redundant side of the hyperplan lead to smaller probabilities for the redundant label. LibSVM rescaled these distances and then transformed them by a sigmoid function into probabilistic measures.

In preliminary testing, we used two other non-linear methods, Bayesian networks and MultilayerPerceptron. In the case of Bayesian network (a generative model), the performance degraded when we tried MaxNrOfParent > 1, which relaxed the number of parents and learned more complicated network structures (data not shown). In the case of the neural network implemented by Weka's MultilayerPerceptron (a non-linear classifier), the performance was worse than SVM regardless of the number of hidden layers (data not shown). Similarly, Radial Basis Function kernel in SVM and the Stacking meta-algorithm did not provide better performance possibly due the same limitation on the training size. Thus, non-linear methods did not appear to provide increased performance in this problem and increased opportunities to overfit the data. This might be due to the large number of attributes (43) but relatively fewer training instances (368), as in [59].

Other pre-processing steps included removing gene pairs with missing values instead of substituting their values with the averages. In addition, highly correlated attributes were also removed because some machine learning algorithms (such as SVM) assume independence among attributes.

### SVM Sensitivity Analysis

We used Pearson correlation of the predicted probabilities before and after removing single attributes to quantify the sensitivity of single attributes when they were removed during the machine learning process. First, a smaller subset of attributes were selected, as described in [60], to ensure they are both informative (by finding attributes that maximize the correlation between them and the redundancy label) and independent (by minimizing the inter-correlations among the selected attributes). This step was necessary because the original set of attributes contained redundant information, so removing any one of them was compensated by other attributes and didn't change the predictions significantly. We used SVM to make predictions using this smaller subset of attributes (19) and then compared with the predictions where each of the attributes was removed from the subset in turn (Additional file 5).

### Description of Information Gain Ratio used on single attribute classifier

Binary partitioning a single attribute by setting a fixed threshold value is the most straightforward classification. Every gene pair with a greater attribute value can thus be predicted redundant (or non-redundant), with the predicted probability corresponding to the ratio of redundant (or non-redundant) pairs over the whole training set. We determined this threshold value by exhaustively testing each possible value of the attribute and kept the one with the maximum information gain ratio to the known label. C4.5 uses the same strategy to select and branch on the attribute iteratively.

### The Withholding Strategy

We used 10-fold stratified cross-validation to evaluate the performance of machine learning algorithms. The original training set was first partitioned into 10 equal-sized subsets. For each fold, a different subset was evaluated using the model learned from the other subsets. The overall performance measures were tallied among all folds; therefore, the method evaluates every instance in the training set. This procedure essentially reduces the variation in estimating the performance by averaging out the bias caused by particular instances. The stratified sampling procedure also reduces the variation by ensuring that the proportion of instances with different labels in each bin is the same as the whole training set. We used two measures for evaluation: recall rate of a particular label is the ratio of true positives over all known positives, and, precision rate is the ratio of true positives over both true positives and false positives.

### Gene Ontology (GO) Analysis

For analysis of over-represented GO terms among redundant and non-redundant genes, genes were split into redundant or non-redundant sets for each size class (large, medium or small if the number of closely related genes are >20, between 5 and 20, or < 5, repectively, using BLAST cutoff of 1e-4). This meant that large gene families were sometimes broken up into more than one paralogous group, depending on how many closely related genes they had. We calculated overrepresented GO terms for cellular component, biological process and molecular function classification systems and then merged results. We then asked what GO terms were over represented (P < 10^-2^) in each set for each size class. GO terms or their descendents were used. We used Bioconductor's GOstats package [61,62] to find the overrepresented GO terms, which derives p-values of over-represented GO terms based on the hypergeometric distribution. We then examined average attributes for genes in each set that mapped to over-represented categories.

## List of abbreviations

SVM: Support Vector Machine; ROC: Receiver Operating Characteristic; AUC: area under the curve; TAIR: The *Arabidopsis *Information Resource; NASC: Nottingham Arabidopsis Stock Centre; GO: Gene Ontology

## Authors' contributions

KDB and DES conceived and designed the project. HWC performed the analysis and participated in the design. SB performed preliminary analysis on the data. DES supervised the computational aspects of the project and KDB supervised the biological aspects of the project. KDB and HWC wrote the manuscript. All authors read and approved the final manuscript.

## Supplementary Material

Additional file 1 **Attribute characteristics of the redundant and non-redundant training sets**. Frequency distribution of redundant vs. non-redundant pairs in the training set grouped by (a) BLAST E-value (b) Pearson correlation of gene pairs in expression profiles across the category "All Experiments." Click here for file

Additional file 2 **A table of precision and recall rates for various probability thresholds**. Click here for file

Additional file 3 **Trend in redundancy calls at varying probability thresholds**. The percentage of all gene pairs tested that were classified as redundant at different probability thresholds Click here for file

Additional file 4 **The synonymous substitution rates (Ks) of redundant and non-redundant training sets**. Frequency distribution of redundant v.s. non-redundant pairs in the training set grouped by intervals of Ks values. Click here for file

Additional file 5 **A table quantifying contributions of attributes toward redundancy predictions**. Click here for file

Additional file 6 **A table of functional trends of redundant or non-redundant genes in various sizes of paralog groups**. Click here for file

Additional file 7 **A table of gene family sizes for each of the over-represented GO terms**. Click here for file

Additional file 8 **Duplication origins of paralogous gene pairs**. Frequency distribution of large-scaled duplication events (recent and old), as well as single and tandem duplications grouped by (a) Synonymous Substitution Rates (Ks) (b) Pearson correlation of gene pairs in expression profiles across the category "All Experiments". Click here for file

Additional file 9 **The training set used by SVM.** The training set includes 97 redundant pairs (class = plus), and 271 non-redundant ones (class = minus). Each line includes 43 pair-wise attributes and the redundancy class for a gene pair. Click here for file

Additional file 10 **The redundancy predictions generated by SVM**. Click here for file
