# Supplementary figures and images for: Predicting genome-wide redundancy using machine learning
Source: BMC Evol Biol. 2010 Nov 18;10:357. doi: 10.1186/1471-2148-10-357 (PMC2998534; doi:10.1186/1471-2148-10-357)

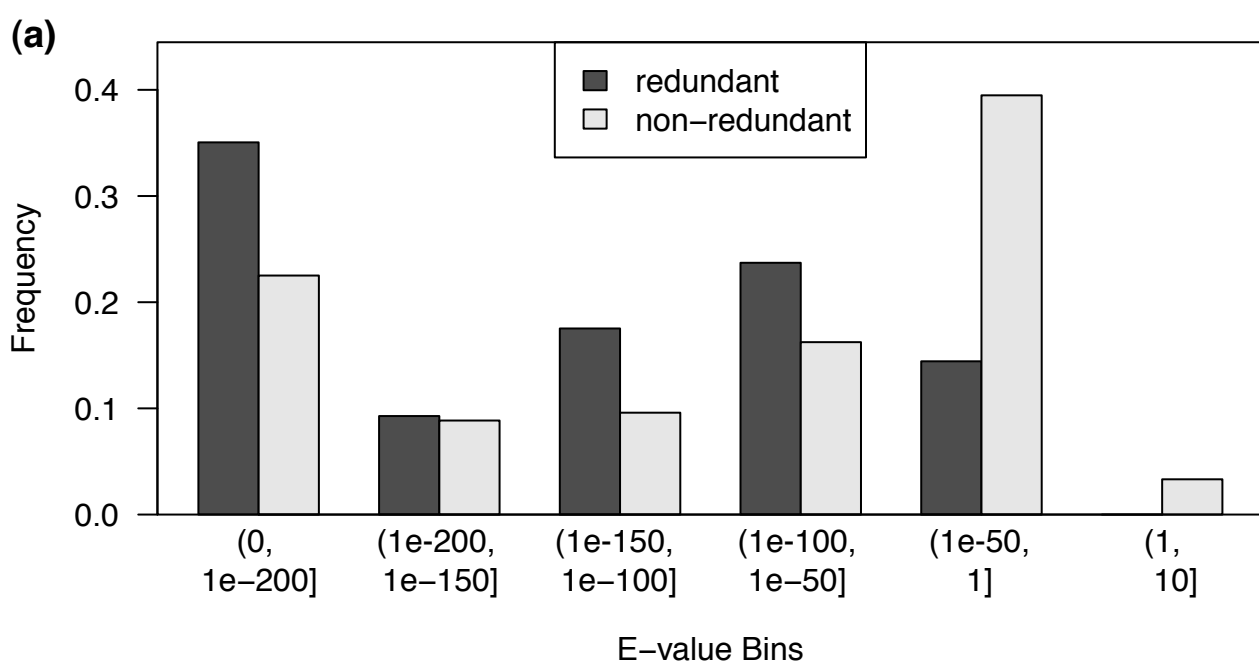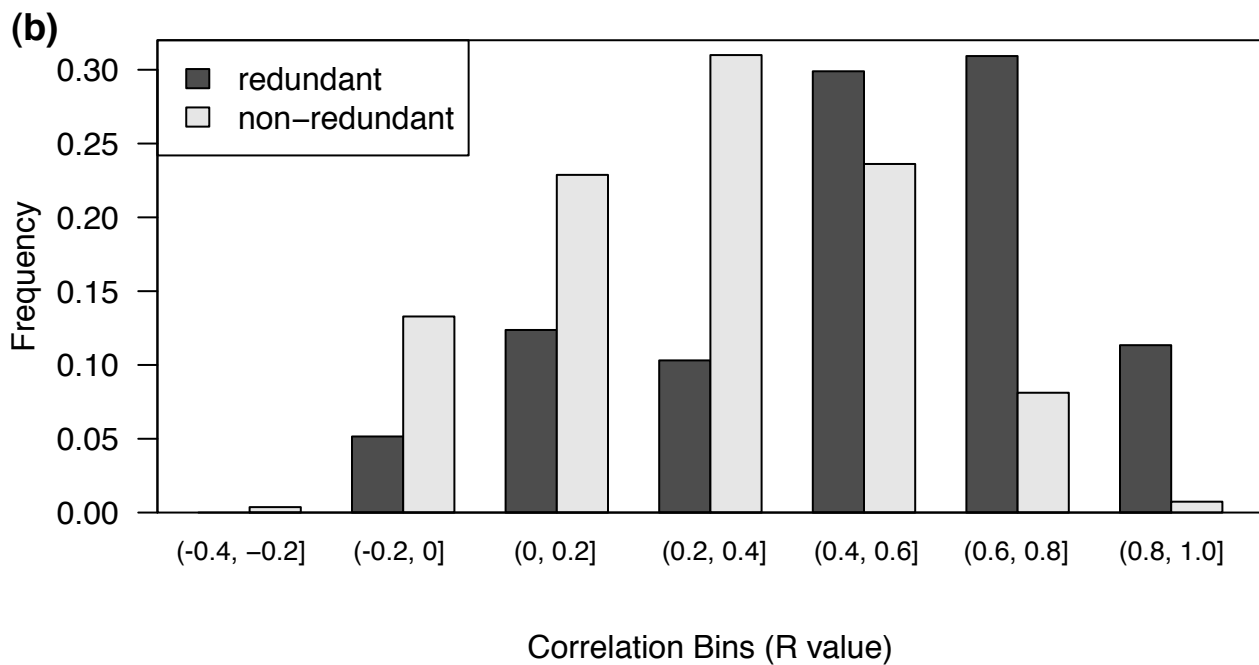

Supplement: Additional file 1 — Attribute characteristics of the redundant and non-redundant training sets. Frequency distribution of redundant vs. non-redundant pairs in the training set grouped by (a) BLAST E-value (b) Pearson correlation of gene pairs in expression profiles across the category "All Experiments." [file 1471-2148-10-357-S1.PDF]

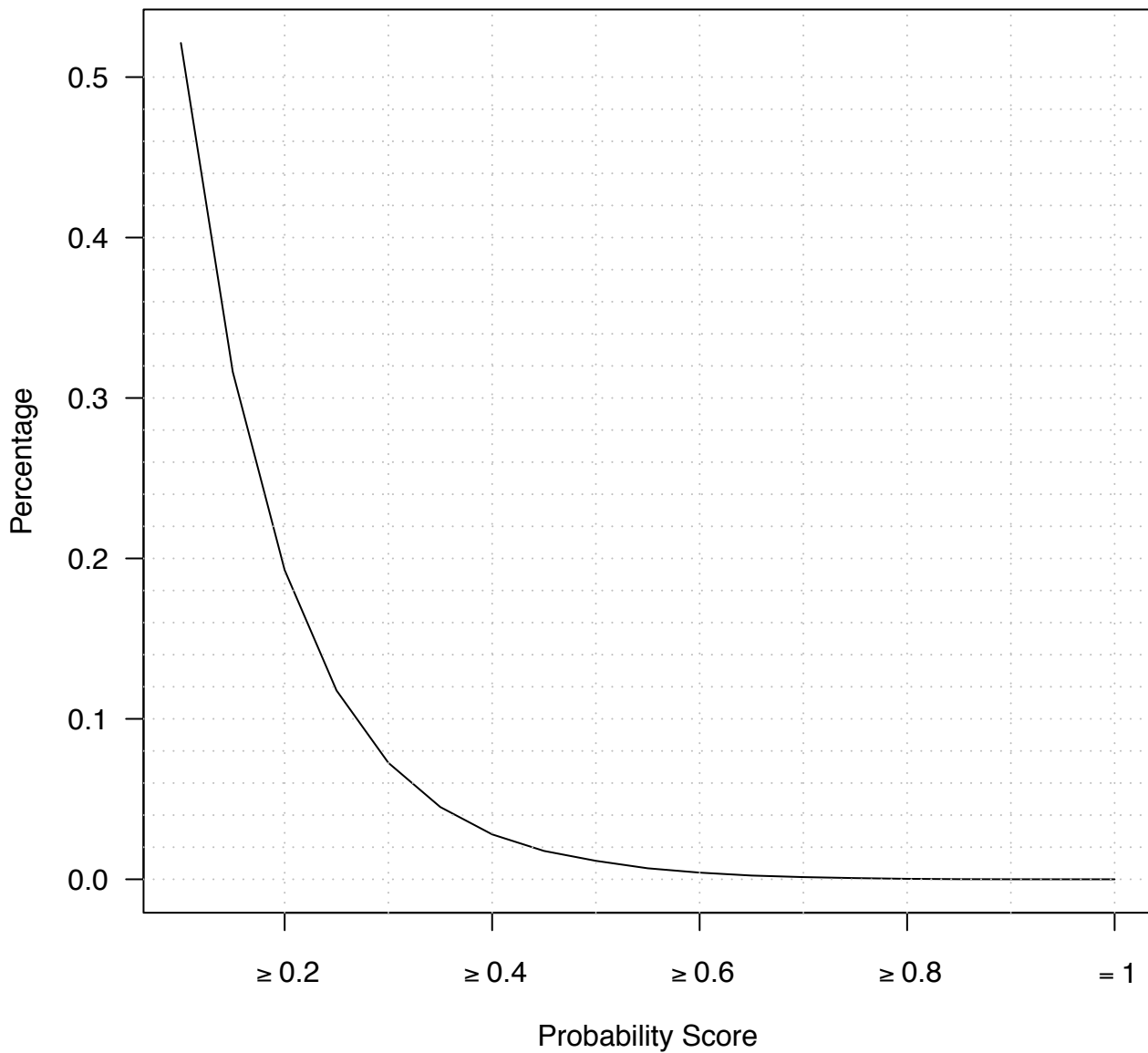

Supplement: Additional file 3 — Trend in redundancy calls at varying probability thresholds. The percentage of all gene pairs tested that were classified as redundant at different probability thresholds [file 1471-2148-10-357-S3.PDF]

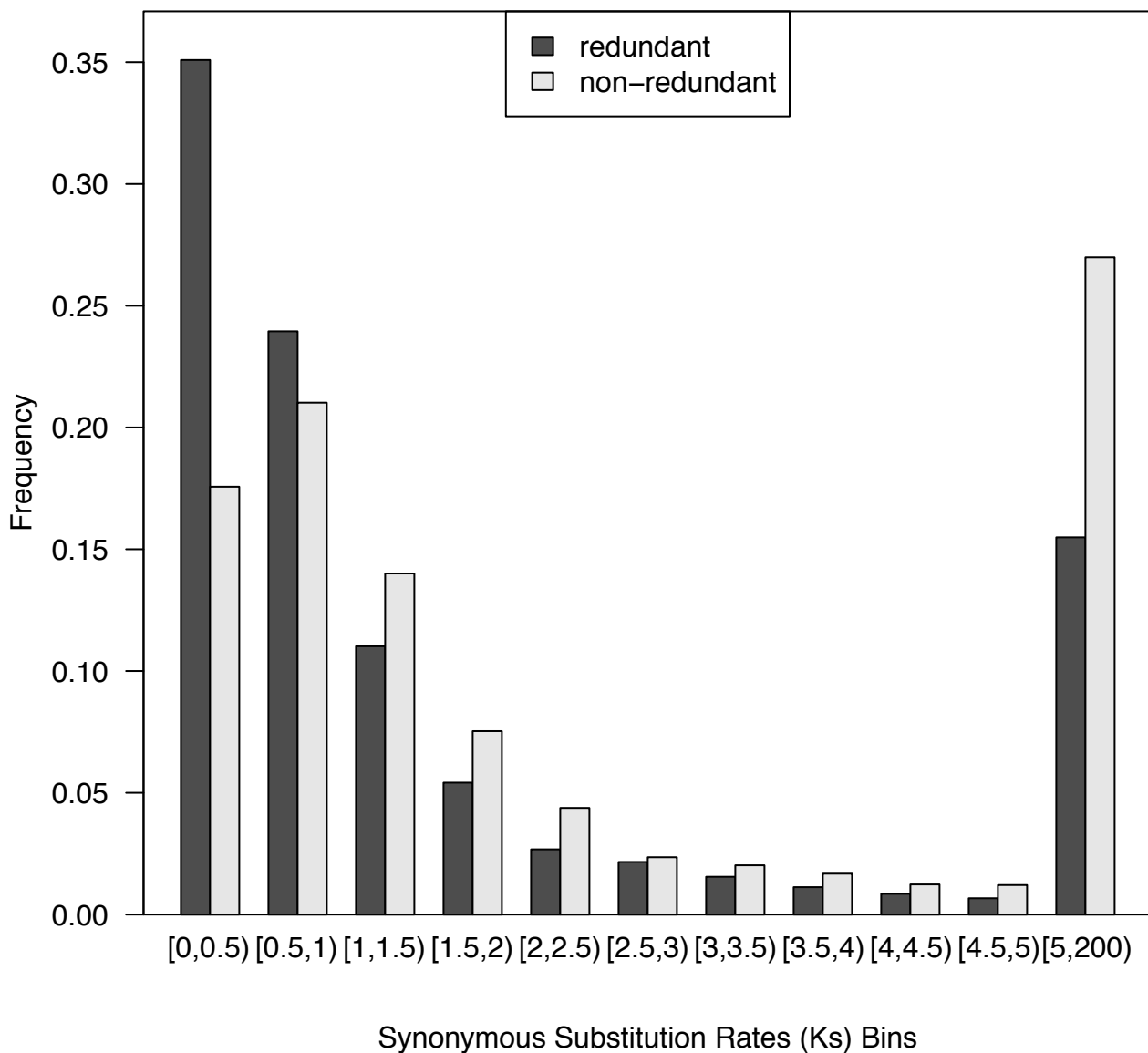

Supplement: Additional file 4 — The synonymous substitution rates (Ks) of redundant and non-redundant training sets. Frequency distribution of redundant v.s. non-redundant pairs in the training set grouped by intervals of Ks values. [file 1471-2148-10-357-S4.PDF]

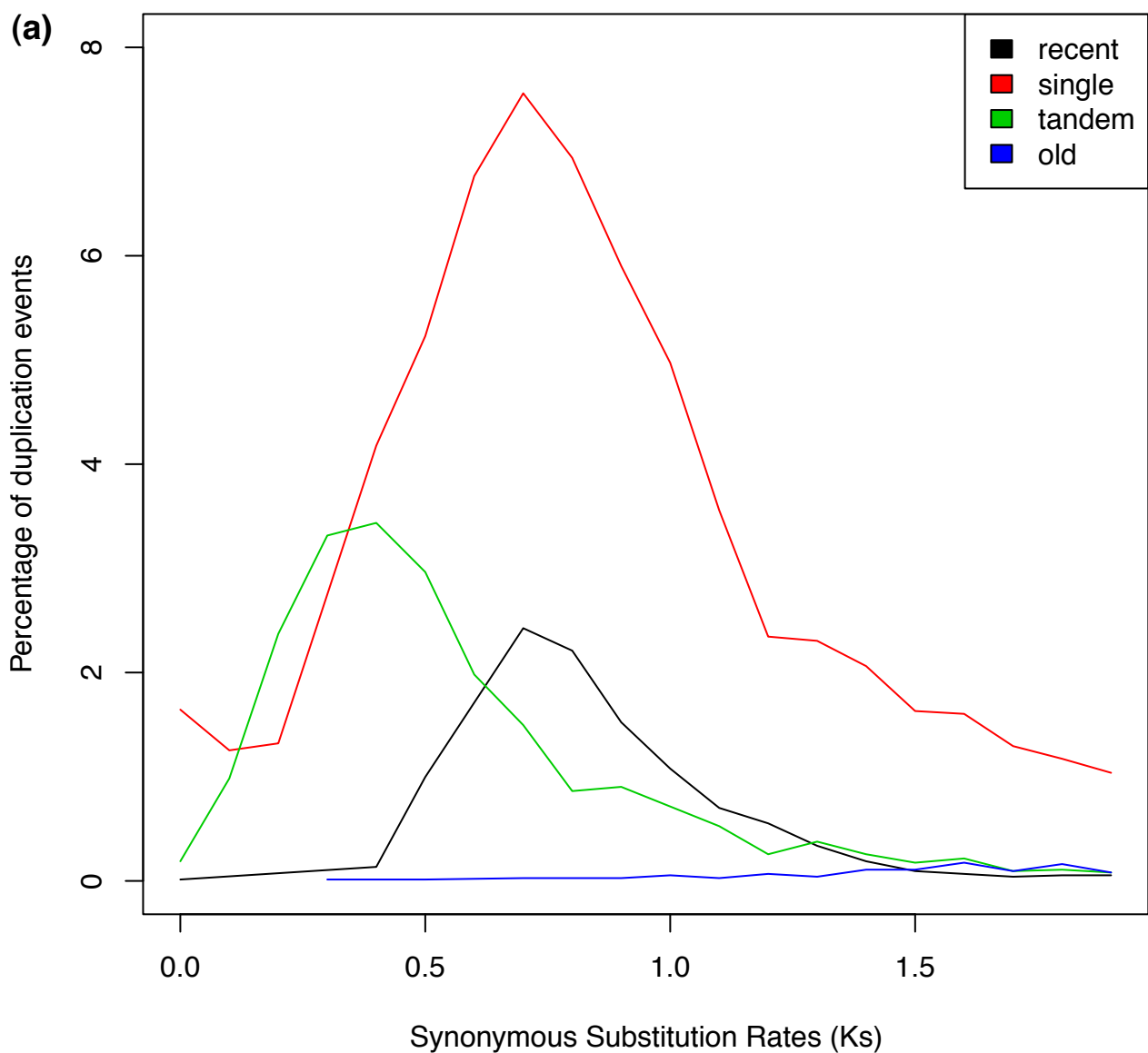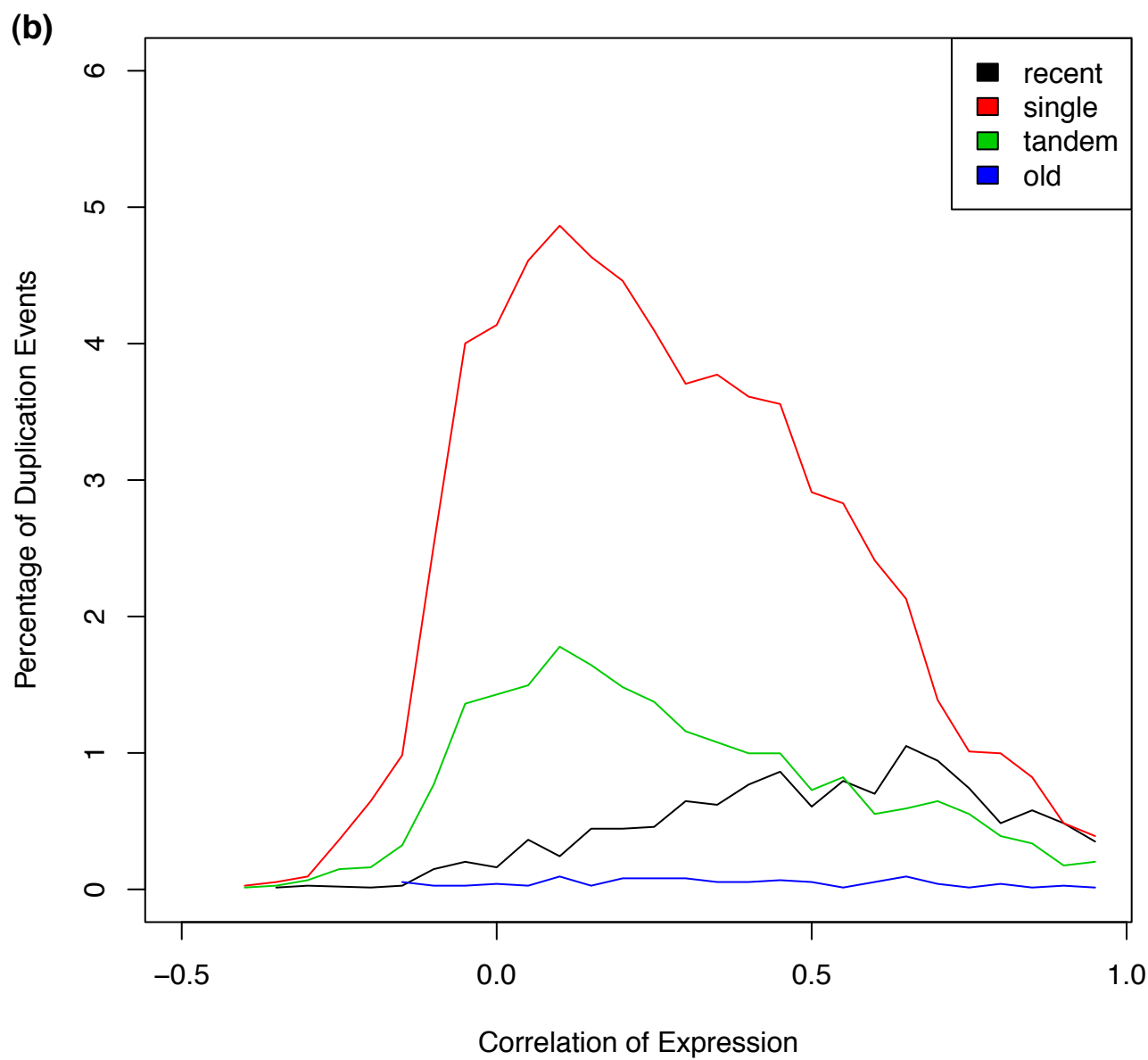

Supplement: Additional file 8 — Duplication origins of paralogous gene pairs. Frequency distribution of large-scaled duplication events (recent and old), as well as single and tandem duplications grouped by (a) Synonymous Substitution Rates (Ks) (b) Pearson correlation of gene pairs in expression profiles across the category "All Experiments". [file 1471-2148-10-357-S8.PDF]
