# Supplementary material for: Predicting genome-wide redundancy using machine learning
Source: BMC Evol Biol. 2010 Nov 18;10:357. doi: 10.1186/1471-2148-10-357 (PMC2998534; doi:10.1186/1471-2148-10-357)
Supplement: Additional file 2 — A table of precision and recall rates for various probability thresholds. [file 1471-2148-10-357-S2.DOC]

Precision and recall rates for different probability thresholds

| **Probability Threshold** | **Recall** | **Precision** |
| --- | --- | --- |
| 0.2 | 0.79 | 0.4 |
| 0.21 | 0.78 | 0.4 |
| 0.22 | 0.75 | 0.41 |
| 0.23 | 0.74 | 0.42 |
| 0.24 | 0.72 | 0.43 |
| 0.25 | 0.7 | 0.44 |
| 0.26 | 0.69 | 0.45 |
| 0.27 | 0.67 | 0.46 |
| 0.28 | 0.65 | 0.47 |
| 0.29 | 0.64 | 0.49 |
| 0.3 | 0.62 | 0.51 |
| 0.31 | 0.61 | 0.52 |
| 0.32 | 0.6 | 0.55 |
| 0.33 | 0.59 | 0.55 |
| 0.34 | 0.56 | 0.56 |
| 0.35 | 0.55 | 0.56 |
| 0.36 | 0.54 | 0.58 |
| 0.37 | 0.52 | 0.58 |
| 0.38 | 0.51 | 0.6 |
| 0.39 | 0.49 | 0.61 |
| 0.4 | 0.48 | 0.62 |
| 0.41 | 0.47 | 0.63 |
| 0.42 | 0.46 | 0.64 |
| 0.43 | 0.45 | 0.65 |
| 0.44 | 0.43 | 0.65 |
| 0.45 | 0.41 | 0.66 |
| 0.46 | 0.4 | 0.67 |
| 0.47 | 0.38 | 0.68 |
| 0.48 | 0.37 | 0.68 |
| 0.49 | 0.35 | 0.7 |
| 0.5 | 0.35 | 0.7 |
| 0.51 | 0.33 | 0.71 |
| 0.52 | 0.31 | 0.71 |
| 0.53 | 0.29 | 0.72 |
| 0.54 | 0.27 | 0.72 |
| 0.55 | 0.25 | 0.72 |
| 0.56 | 0.24 | 0.74 |
| 0.57 | 0.23 | 0.74 |
| 0.58 | 0.21 | 0.74 |
| 0.59 | 0.2 | 0.74 |
| 0.6 | 0.19 | 0.76 |
